# Oral health-related quality of life in patients with cleft lip and/or palate or Robin sequence

**DOI:** 10.1007/s00056-022-00414-6

**Published:** 2022-07-19

**Authors:** D. Payer, M. Krimmel, S. Reinert, B. Koos, H. Weise, C. Weise

**Affiliations:** 1 Department of Orthodontics, University Hospital Tuebingen, Osianderstr. 2–8, 72076 Tuebingen, Germany; 2 Department of Oral and Maxillofacial Surgery, University Hospital Tuebingen, Osianderstr. 2–8, 72076 Tuebingen, Germany

**Keywords:** Retrognathia, Glossoptosis, Upper airway obstruction, Craniofacial abnormalities, Child Oral Health Impact Profile, Retrognathie, Glossoptose, Obstruktion der oberen Atemwege, Kraniofaziale Anomalien, Child Oral Health Impact Profile

## Abstract

**Purpose:**

To compare the oral health-related quality of life (OHRQoL) in patients with cleft lip and/or palate or Robin sequence versus a healthy control group using the Child Oral Health Impact Profile (COHIP-G19). Factors such as age, gender, and cleft type were considered.

**Methods:**

Over an 8-month period, the OHRQoL was surveyed by using the COHIP-G19 questionnaire. Included were patients with a craniofacial disorder (*n* = 61; average age 11.24 years) and a healthy control group (*n* = 70, average age 12.63 years) for a total of 131 patients (average age 11.99 years) from the Department of Orthodontics University Hospital Tübingen, Germany. These were divided into two age groups (6–11 years; 12–18 years).

**Results:**

Statistically, patients with a craniofacial disorder presented a significantly lower OHRQoL than the control group (*p* = 0.0055). In the craniofacial disorder group, older patients revealed a significantly (*p* = 0.005) lower OHRQoL than the younger patients. Female patients showed in nearly all groups a better OHRQoL than male patients, but this difference was not statistically significant (*p* > 0.05). Males with a craniofacial disorder scored significantly lower than males without (*p* = 0.016); females showed no differences between the groups. Visibility, location, and severity of the craniofacial malformation did not have a significant influence on the OHRQoL.

**Conclusion:**

The occurrence of a craniofacial malformation impacted the OHRQoL especially in older and male affected patients, unrelated to the expression level or localization. An early instruction about oral health, rehabilitation and functional training should be considered in therapy.

## Abstract (de)

**Ziel:**

Vergleich der mundgesundheitsbezogenen Lebensqualität (OHRQoL) von Patienten mit Lippen- und/oder Gaumenspalte oder Robin-Sequenz mit einer gesunden Kontrollgruppe anhand des Child Oral Health Impact Profile (COHIP-G19). Faktoren wie Alter, Geschlecht und Spaltentyp wurden berücksichtigt.

**Methoden:**

Über einen Zeitraum von 8 Monaten wurde die OHRQoL mit Hilfe des COHIP-G19-Fragebogens erhoben. Eingeschlossen wurden Patienten mit einer kraniofazialen Anomalie (*n* = 61; Durchschnittsalter 11,24 Jahre) und eine gesunde Kontrollgruppe (*n* = 70, Durchschnittsalter 12,63 Jahre), insgesamt also 131 Patienten (Durchschnittsalter 11,99 Jahre) aus der Abteilung für Kieferorthopädie des Universitätsklinikums Tübingen, Deutschland. Diese wurden in 2 Altersgruppen eingeteilt (6–11 und 12–18 Jahre).

**Ergebnisse:**

Statistisch gesehen wiesen die Patienten mit einer kraniofazialen Störung eine signifikant niedrigere OHRQoL auf als die Kontrollgruppe (*p* = 0,0055). In der Gruppe der Patienten mit kraniofazialen Störungen wiesen ältere Patienten eine statistisch signifikant (*p* = 0,005) niedrigere OHRQoL auf als die jüngeren. Weibliche Patienten zeigten in fast allen Gruppen eine bessere OHRQoL als männliche Patienten, aber dieser Unterschied war statistisch nicht signifikant (*p* > 0,05). Jungen mit einer kraniofazialen Störung schnitten signifikant schlechter ab als Jungen ohne diese Störung (*p* = 0,016); bei den Mädchen gab es keine Unterschiede zwischen den Gruppen. Sichtbarkeit, Lage und Schweregrad der kraniofazialen Fehlbildung hatten keinen signifikanten Einfluss auf die OHRQoL.

**Schlussfolgerung:**

Vor allem ältere und männliche Patienten mit einer kraniofazialen Fehlbildung hatten eine niedrigere OHRQoL, unabhängig von der Ausprägung oder Lokalisation. Eine frühzeitige Aufklärung über Mundgesundheit, Rehabilitation und Funktionstraining sollte in der Therapie berücksichtigt werden.

## Introduction

Cleft lip and/or palate (CL/P) is the most common malformation occurring in approximately 1:600 live births [18]. Cleft malformation can arise in different combinations, in varying degrees of severity as well as visibility of the cleft, such as uni- (U) or bilateral (B) CL/P, cleft palate only (CP) or cleft lip with or without alveolus (CL ± A).

A craniofacial malformation that is associated in 80–90% of the cases with a CP is the Robin sequence (RS) [16, 49]. The prevalence of RS is 11.3:100,000 of live births [46]. This malformation involves the triad of mandibular retrognathia, glossoptosis, and resultant upper airway obstruction [61, 62]. Patients with CL/P as well as RS exhibit severe functional difficulties such as feeding problems and failure to thrive in the first weeks after birth. Duration and intensity of interdisciplinary rehabilitation therapy that includes neonatologists, craniomaxillofacial surgeons, ear, nose and throat specialists, speech therapists, orthodontists, and psychologists depend on the severity of the craniofacial malformation. Therapy begins at birth and can last until adolescence. Furthermore, hearing and speech development are also strongly influenced by a cleft in the soft palate. Problems with oral hygiene, missing or malpositioned teeth, arch form deformation, oro-nasal fistulas, nasal deformity, lip scar, facial appearance, and distinctive skeletal discrepancies between the lower and upper jaw and velopharyngeal insufficiencies are additional concerns that affect therapy.

These patients do not only present physical challenges. The malformation can also influence comprehension, cognition, and communication [33]. These in turn impact well-being, self-esteem and eventually the psyche of patients, thus, affecting social life, social interaction, and quality of life (QoL) [1, 39, 40, 45, 57]. In the current literature, we found only two studies on the QoL of RS patients and none comparing them with nonsyndromal CL/P patients [8, 21].

In a society dominated by improved living conditions, beauty ideals, and personal well-being, the term QoL has become increasingly emphasized in many areas of science [29]. In medicine, QoL was introduced in 1975 [7]. Topolsky et al. showed a difference in QoL in adolescents with facial conspicuity [71]. In dentistry, QoL and its impact on health has only recently been considered relevant. Reisine et al. were the first to demonstrate the importance of QoL in relation to oral disease [60]. In 2003, the World Health Organization (WHO) recognized oral health-related quality of life (OHRQoL) as a segment of the Global Oral Health Program [56]. OHRQoL is more specific than QoL as a factor in determining the functional and psychosocial implications emanating from oral diseases [44]. The effects of these diseases impact social life, functional well-being, satisfaction, and expectations concerning care of the afflicted persons and their caregivers [66, 76].

In contrast to the clinical assessment of oral health by dentists, John et al. described this measure as revealing how patients themselves assess the status of their oral health [35], including factors such as orofacial function, pain, appearance, and psychosocial effects [35, 36]. These in turn have significant implications for everyday clinical practice and dental research. The need for scales to measure OHRQoL has therefore been growing in the last 20 years in dentistry [66]. In 1976, Cohen developed sociodental indicators [19], which led to the development of instruments for measuring OHRQoL [40, 50, 67].

To assess OHRQoL, validated and standardized questionnaires are mandatory [9]. Various questionnaires have been developed by several authors and tested for their psychometric properties. The most frequently used questionnaire for children and adolescents is the Child Oral Health Impact Profile (COHIP) questionnaire [26, 27]. Broder et al. designed the original COHIP questionnaire to assess the self-reported OHRQoL of children and adolescents aged 8–15 years. It was subsequently adjusted to the ages of 7–18 years for easier handling by patients and for better comparison of the results between age groups [12, 14]. The authors even published a short version of 19 questions of the original 34-question COHIP questionnaire that provides similar results regarding reliability, validity, and sensitivity of the data. Because of its broad applicability and coverage of psychometric properties, the COHIP-19 questionnaire is best suited for assessing OHRQoL [15, 22, 26].

The aim of this study was to evaluate a possible difference in OHRQoL between and within a group of patients with craniofacial disorders and a control group, using the COHIP-G19 questionnaire. Factors such as age, gender, visibility of the cleft, cleft type, as well as the severity of the malformation were also considered. The following null hypotheses were investigated:

1. Patients with craniofacial disorders do not differ in OHRQoL compared with a healthy reference group.
2. There is no age-related difference in OHRQoL in either group.

## Methods

### Study design

This exploratory cross-sectional study was designed to be prospective and monocentric at the Department of Orthodontics at Tübingen University Hospital. During a routine follow-up visit, the patients were invited to participate in the study by a clinician directly involved in their orthodontic care. All patients and their legal guardians were informed both verbally and by means of a written information sheet in advance that their participation was voluntary. They also were informed of the whole procedure and the aim of the study, as well as about the pseudonymized data collection. A consent form for the patient in the study was signed by at least one parent or caregiver prior to data collection. All examinations were noninvasive, not stressful for the participants, and could be carried out in one session, within about 20 min. Patients with incomplete questionnaires or missing consent were excluded from the evaluation. This study was approved by the institutional ethics committee of Tübingen University Hospital (approval number: 188/2019BO1).

### Patients

Over an 8‑month period, 131 patients were recruited for this study. All patients received orthodontic treatment in our department. Patients with craniofacial disorders such as RS and all variations of CL/P were included. They were treated in our interdisciplinary center with a well-known therapy concept and underwent reconstructive cleft surgery [75, 77, 78]. Patients unaffected by craniofacial malformation were selected by the treating orthodontist in our department. Exclusion criteria for participation in this study were defined as additional complex congenital malformations (syndromes), psychological limitations, general illnesses, and nonmastery of the German language. The patients were divided into two groups:

- Group 1 with craniofacial malformation (cranio) and
- Group 2 without craniofacial disorders (control).

These two groups were additional divided into age groups of 6–11 years and 12–18 years. Puberty, which is associated with more self-reflective and awareness during adolescence, was used to divide the two groups, i.e., between 11 and 12 years [25].

### Instruments

The German-translated short form of the COHIP-G19 questionnaire was used for a self-report measure of the OHRQoL [64]. COHIP-G19 consists of 19 questions divided into three subcategories: oral health/well-being, functional well-being, and social-emotional/school/self-image aspects. Each question asks how often patients had negative or positive experiences in the last 3 months. The total of 19 questions of the COHIP-G19 could be answered with “never”, “almost never”, “sometimes”, “quite often” or “almost always.” The patient only had to put a cross in the corresponding box. Questionnaires that were not completed in full or in which individual questions were not answered were excluded from the evaluation. The different answer options were scored using a different number of points. These scores for the three subcategories were added together to give an overall score, i.e., the COHIP-G19 score. The COHIP-G19 score can vary from 0 points (the worst OHRQoL) to 76 points (the best OHRQoL). We interpreted the responses as follows: the higher the COHIP-G19 score, the better the oral health-related quality of life of the respective patient.

### Statistical data analyses

Patient data were collected from our electronic database, clinical records, and pseudonymized form and saved in an excel sheet (Microsoft®, Redmond, WA, USA). Statistical evaluation and descriptive statistics were performed using JMP (version 15.2.0, SAS Institute Inc., Cary, NC, USA). The COHIP score calculation was quantified by average, minimum and maximum values, and standard deviations. To evaluate the internal consistency of the COHIP-G19 questionnaire, we used the test score reliability coefficient Cronbach’s α. Test–retest reliability of COHIP in German was made with the interclass correlation coefficient (ICC). COHIP total score and three subscale scores were applied using statistical analyses of variance between group 1 (craniofacial malformation) and group 2 (control) factoring in gender (male vs. female) and age group (6–11 years vs. 12–18 years), cleft distribution, visibility and all 2‑way interactions as independent variables. Statistical significance using parametric paired sample pooled t‑test was considered at *p* < 0.05.

## Results

### Characteristics of patients

**Fig. 1:**
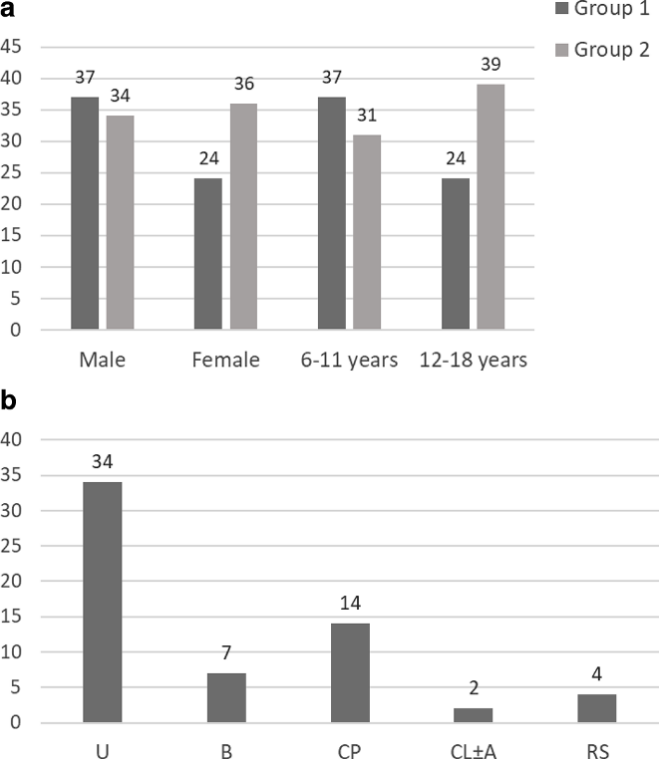
**a** Distribution of participants in gender (male/female) and age groups (6–11 years/12–18 years). **b** Distribution of craniofacial disorders (group 1). *CP* cleft palate, *CL* *±* *A* cleft lip with or without alveolus, *RS* Robin sequence, *U* Unilateral CL/P, *B* Bilateral CL/P **a** Verteilung der Teilnehmenden nach Geschlecht (männlich/weiblich) und Altersgruppen (6-11 Jahre/12-18 Jahre). **b** Verteilung der kraniofazialen Anomalie (Gruppe 1). *CP* Gaumenspalte, *CL* *±* *A* Lippenspalte mit oder ohne Alveole *RS* Robin-Sequenz, *U* CL/P einseitig, *B* CL/P beidseitig

The characteristics of all patients who participated in this study are presented in Table 1 and Fig. 1. In total, 131 patients (60.66% male, 39.34% female) were included here. They were divided into two groups: group 1, those with a craniofacial disorder (*n* = 61), and group 2, the controls (*n* = 70). The age groups were divided into 6–11 year olds (68 patients) and 12–18 year olds (63 patients). The average age was 11.98 ± 3.28 years. In group 2, one patient was excluded because of incomplete data. This patient did not answer one question and was not included in the statistical evaluation of the study.

**Table 1:** Characteristics and distribution of patients in group 1 (craniofacial malformation) and group 2 (control) Merkmale und Verteilung der Patienten in Gruppe 1 (kraniofaziale Fehlbildung) und Gruppe 2 (Kontrollen)

	Group 1 *n* = 61		Group 2 *n* = 70	
	*n*	%	*n*	%
*Age*				
Mean	11.24	–	12.63	–
SD	3.19	–	3.20	–
*Gender*				
Male	37	60.66	34	48.57
Female	24	39.34	36	51.43
*Craniofacial disorder*				
U	34	55.74	–	–
B	7	11.48	–	–
CP	14	22.95	–	–
CL ± A	2	3.28	–	–
RS	4	6.56	–	–
*Cleft side*	36	–	–	–
Left side	28	77.70	–	–
Right side	8	22.20	–	–
*Distribution age group*				
6–11 years	37	60.66	31	44.28
12–18 years	24	39.34	39	55.71

*CP* cleft palate, *CL* *±* *A* cleft lip with or without alveolus, *RS* Robin sequence, *U* Unilateral CL/P, *B* Bilateral CL/P, *SD* standard deviation

### Reliability analysis of COHIP-G19 questionnaire

Test–retest reliability of COHIP in German had an interclass correlation coefficient (ICC) of 121. Table 2 presents the test score reliability coefficient, or Cronbach’s α, for the total COHIP-G19 questionnaire of group 1 (with craniofacial malformation) and group 2 (control). Internal consistency was measured as good for nearly all subscales. Unacceptable values in internal consistency were found in the functional (0.37) and socioemotional (0.48) subscales of group 2. The test for group 1, the patients with craniofacial anomalies, seemed to present a higher range of reliability for the COHIP-G19 questionnaire than the control group 2. All values of Cronbach’s α were higher in group 1, except for the oral-health subscale (group 1: 0.52; group 2: 0.55).

**Table 2:** Cronbach’s α values for group 1 (craniofacial malformation) and 2 (control) of the COHIP total score and subscale scores Cronbachs α‑Werte für Gruppe 1 (kraniofaziale Fehlbildung) und 2 (Kontrolle) des COHIP-Gesamtergebnisses und der Unterskalenwerte

		Cronbach’s α (*n* = 131)	
	Number of items	Group 1 *n* = 61	Group 2 *n* = 70
COHIP Total subscale	19	0.62	0.57
*Subscale*			
Oral-health	5	0.52	0.55
Functional	4	0.50	0.37
Socioemotional	10	0.59	0.48

*COHIP* Child Oral Health Impact Profile questionnaire

### Analysis of the COHIP-G19 questionnaire

Table 3 presents the descriptive analysis of the COHIP total scores, subscale total scores and scores of the 19 questions for both groups. The COHIP total score of group 1 (average 57.77 points) versus group 2 (control; average 62.85 points) showed a statistically significant difference of 5 points (*p* = 0.005). The oral-health subscale showed a significantly higher (*p* = 0.001) average score in control group 2 (15.27 points) as compared to group 1 (13.46 points). In the functional subscale, question eight (“had difficulty saying certain words”) revealed a significant difference (*p* = 0.004) between both groups with a higher average value in group 2 (3.34 points). In the socioemotional subscale, the subpoints 15, “been bullied,” (*p* = 0.00) and 18, “been confident,” (*p* = 0.01) showed significantly higher COHIP score values in group 2.

**Table 3:** Descriptive analysis of COHIP total score and subscale scores of group 1 (craniofacial malformation) and group 2 (control) Deskriptive Analyse des COHIP-Gesamtergebnisses und der Unterskalenwerte von Gruppe 1 (kraniofaziale Fehlbildung) und Gruppe 2 (Kontrolle)

	Group 1 *n* = 61		Group 2 *n* = 70			
	Mean	SD	Mean	SD	*F* ratio	*P* value
COHIP total	57.77	11.66	62.85	8.33	0.004	0.005*
Subscale						
*Oral-health total*	*13.46*	*3.40*	*15.27*	*2.69*	*0.001*	0.001*
1. Had pain in your teeth	3.08	0.82	3.26	0.75	1.613	0.206
2. Had discolored teeth	2.95	1.26	3.34	0.99	3.970	0.048*
3. Had crooked teeth/space between your teeth	1.47	1.50	2.20	1.26	9.032	0.003*
4. Had bad breath	3.09	1.16	3.26	0.88	0.786	0.377
5. Had bleeding gums	2.85	1.08	3.21	0.90	4.390	0.040*
*Functional total*	*12.578*	*3.06*	*13.36*	*2.78*	*0.128*	*0.130*
6. Had difficulty in eating	3.26	1.06	3.34	1.12	0.010	0.919
7. Had trouble sleeping	3.62	0.82	3.60	0.84	0.025	0.975
8. Had difficulty saying certain words	2.75	1.30	3.34	0.99	8.620	0.004*
9. Had difficulty keeping your teeth clean	2.93	1.12	3.17	0.95	1.710	0.193
*Socioemotional total*	*31.74*	*7.65*	*34.23*	*5.31*	*0.031*	*0.035*
10. Been unhappy or sad	3.15	1.21	3.17	1.06	0.014	0.904
11. Felt worried or anxious	3.46	0.94	3.67	0.65	2.290	0.132
12. Avoided smiling or laughing with others	3.24	1.12	3.57	0.86	3.520	0.063
13. Felt that you look different	2.75	1.52	3.04	1.42	1.260	0.260
14. Been worried about what other people think about your teeth/mouth/face	2.23	1.50	2.46	1.41	0.800	0.370
15. Been bullied	3.28	1.10	3.81	0.62	12.202	0.000*
16. Missed school for any reasons	3.69	0.74	3.87	0.59	2.470	0.110
17. Not wanted to speak/read loud in class	3.77	0.53	3.67	0.70	0.820	0.370
18. Been confident	3.05	1.19	3.53	0.91	6.790	0.010*
19. Felt that you were attractive (good looking)	3.11	1.27	3.43	1.00	2.500	0.110

*COHIP* Child Oral Health Impact Profile questionnaire, *SD* standard deviation

*Statistically significant (*p* < 0.05)

Table 4 presents the descriptive analysis of COHIP-G19 total scores and subscale scores for group 1 (the patients with a craniofacial disorder) and for group 2 (controls), divided by gender and age. The average COHIP total score of the younger group, 6–11 years of age (average 61.10 points), was statistically significantly higher (*p* = 0.005) than the total score of the older group, 12–18 years of age (average 52.62 points), in group 1. In this group, the score for the socioemotional subscale was significantly (*p* = 0.001) higher in the younger group with 6–11 years (average 34.30 points) as compared to the group of patients being 12–18 years of age (average 27.79 points). In group 2, those with 12–18 years of age had a lower COHIP score than the younger group, although this finding was not statistically significant in the total COHIP score as well as in the subscales. The age group of 6–11 years had a three-point lower COHIP total score in group 1 (average 61.10 points) than in group 2 (average 64.55 points), which was not statistically significant. The oral-health subscale between group 1 (average 13.89 points) and group 2 (average 15.87 points) showed a statistically significant (*p* = 0.004) two-point difference. The 12- to 18-year-old patients in group 1 (average 52.62 points) scored significantly nine points lower (*p* = 0.000) as compared to group 2 (average 61.51 points). The oral-health (*p* = 0.007) and socioemotional (*p* = 0.000) subscales for this age group showed significantly lower COHIP points in group 1.

**Table 4:** Descriptive analysis of COHIP total score and subscale scores according to age groups and gender Deskriptive Analyse des COHIP-Gesamtergebnisses und der Unterskalenwerte nach Altersgruppen und Geschlecht

	Age group				Gender			
	6–11 years *n* = 68	12–18 years *n* = 63			Male *n* = 71	Female *n* = 60		
	Mean (SD)	Mean (SD)	*F* ratio	*P* value	Mean (SD)	Mean (SD)	*F* ratio	*P* value
*Group 1 (craniofacial malformation)*								
COHIP total	61.10 (10.41)	52.62 (11.82)	8.687	0.005*	56.24 (12.12)	60.13 (10.74)	1.630	0.207
Subscale								
Oral-health	13.89 (3.21)	12.79 (3.66)	1.530	0.221	13.19 (3.32)	13.88 (3.58)	0.585	0.447
Functional	12.92 (2.91)	12.04 (3.28)	1.198	0.278	12.43 (3.13)	12.79 (3.01)	0.198	0.658
Socioemotional	34.30 (6.45)	27.79 (7.79)	12.545	0.001*	30.62 (8.06)	33.46 (6.78)	2.035	0.159
*Group 2 (control)*								
COHIP total	64.55 (9.11)	61.51 (7.51)	2.333	0.131	61.88 (9.29)	63.78 (7.33)	0.902	0.345
Subscale								
Oral-health	15.87 (2.67)	14.79 (2.65)	2.834	0.097	15.50 (2.50)	14.06 (2.88)	0.473	0.494
Functional	13.61 (3.18)	13.15 (2.44)	0.466	0.497	13.03 (3.17)	13.67 (2.37)	0.916	0.342
Socioemotional	35.06 (6.03)	33.56 (4.63)	1.388	0.243	33.35 (5.81)	35.06 (4.72)	1.820	0.182
COHIP total								
F ratio^a^	0.156	0.000	–	–	0.032	0.122	–	–
P value^a^	0.078	0.000*	–	–	0.016*	0.061	–	–

*COHIP* Child Oral Health Impact Profile questionnaire, *SD* standard deviation

*Statistically significant (*p* < 0.05)

^a^Combined for group 1 with 2, comparing first age group and second gender

Regarding gender distribution, the trend suggests that males across all groups had lower COHIP scores as compared to female patients, although this finding was not statistically significant. Male patients from group 1 with a craniofacial disorder (average 56.24 points) scored five points lower, which was statistically significant (*p* = 0.016) in the COHIP total values as compared to group 2. These findings are also reflected in the oral-health subscale (*p* = 0.000). Females in group 1 (average 60.13 points) showed a three-point lower COHIP total score than group 2 (average 63.78 points), but this difference was not statistically significant.

Group 1 of patients with a craniofacial disorder, if they were examined separately, showed no statistically significant results in the COHIP total score depending on the cleft type and visibility (Table 5). Patients with bilateral clefts showed the lowest COHIP total score (average 54.57 points), while patients with a CL ± A showed the highest score (average 60.50 points). Regarding patients with RS, the result showed the second lowest COHIP total score value (average 56.00 points). Patients with a visible cleft (average 57.11) showed a two-point lower COHIP total score as compared to patients with a nonvisible cleft (average 59.21).

**Table 5:** COHIP total score subdivided according to cleft type and visibility of the cleft COHIP-Gesamtscore, unterteilt nach Spalttyp und Sichtbarkeit der Spalte

		Group 1	
	*n*	COHIP total Mean	SD
*Craniofacial disorder*	61	–	–
Unilateral CLP	34	57.41	11.54
Bilateral CLP	7	54.57	18.03
CP	14	60.36	10.26
CL ± A	2	60.50	6.36
RS	4	56.00	8.48
*Visible cleft*	42	57.11	12.54
*Nonvisible cleft*	19	59.21	9.58

*CL/P* cleft lip and/or palate, *CP* cleft palate, *CL* *±* *A* cleft lip with or without alveolus, *RS* Robin sequence, *COHIP* Child Oral Health Impact Profile questionnaire, *SD* standard deviation

## Discussion

Our results show a significantly lower OHRQoL in patients with craniofacial disorders compared to a control group without craniofacial disorders who, though, were receiving orthodontic treatment. The outcome was revealed in a statistically higher COHIP total score of the control group. This finding refutes the first of our null hypotheses. A few studies have been published on the OHRQoL using a COHIP questionnaire in patients with craniofacial disorders or at least CL/P. The results of our study corroborate with those of Aravena et al. [5], Ali et al. [2], Broder and Wilson-Genderson [12], and Ward et al. [76], in terms of a population of American and Chilean children with CL/P. But overall, due to differences in study methods, sample sizes, and standardization, it is difficult to combine the results of previous studies and compare them with our own. Antonarakis et al. [4] evaluated in a review the OHRQoL of nonsyndromic patients with CL/P in comparison to a general noncleft population in children and adults. In 2 of the 3 studies, the OHRQoL was found to be significantly lower in patients with CL/P. In the third study, there were no significant differences between noncleft and cleft populations [4]. The COHIP total for the oral-health subscale showed a significantly 2 points lower value in patients with a craniofacial disorder. Patients with a craniofacial disorder had a lower OHRQoL, especially regarding the question of discolored teeth, crooked teeth, and bleeding gums. Stelzle et al. found a statistically significant correlation between gingival esthetics and OHRQoL in patients with CL/P [69]. In terms of functional well-being, a statistically significantly lower COHIP score was reported in patients with a craniofacial disorder in response to questions on pronunciation and the difficulty saying certain words. These results were consistent with those described in a study from Chetpakdeechit et al. [17] and Aravena et al. [5]. They reported that patients with CL/P felt different because of their speech difficulties. This fact is due to the cleft of the soft palate, which is a very important part of the palate, influencing pronunciation, and the emphasis of certain sounds. If there is a velopharyngeal insufficiency, marked by the soft palate not being closed tightly between the mouth and the nasal cavity, the affected patient will suffer from strong hypernasal resonance and problems with pronunciation. In order to ensure sufficient closure and to facilitate speaking at a young age, the palate is sealed as early as possible in our interdisciplinary center by cleft palate repair following Sommerlad’s technique. In addition, a speech pathologist is consulted for successful rehabilitation [70]. Hypernasal resonance can influence psychological and social factors in the lives of affected patients [32]. According to our study, and regarding the socioemotional subscale, bullying increased and confidence levels were significantly lower in patients with a craniofacial disorder. Berk et al. showed that patients with a CL/P had much lower self-esteem compared to their unaffected siblings [10]. Furthermore, patients with CL/P have reported that their self-confidence had been affected by their disorder [37, 73]. This is in line with the results of our study. There is evidence that patients with craniofacial disorder or those with extreme malocclusion have higher occurrences of being bullied among children and adolescents [72]. This may lead to major psychosocial problems and difficult social relationships [31, 52]. These findings reflect the importance of communication skills and the need for early rehabilitation. A cooperative family environment for patients is especially important here. Relatives play a major role in language learning and offer critical support in the development of a psychologically stable individual. It is important to remember that the occurrence of a craniofacial disorder affects the lives of parents as well as a wider family circle. Research indicates that parents can suffer from depression, anxiety, and psychological distress [42, 43]. This reflects the fact that parents struggle with their own emotions regarding a child’s malformation, its effects on their child’s speech, the social reaction of others, and the concerns regarding cleft treatment [53, 54, 63]. Treatment of the patient is not the only concern, as the initiation of treatment for a craniofacial anomaly begins at a prenatal stage [68]. Early diagnosis, education of the parents, and prenatal counselling can reduce parental anxiety associated with this [48]. Furthermore, providing psychiatric or psychologic counseling and treatment to the parents can prove critical in supporting those with cranial disorders [42, 55]. High levels of positive reinforcement, support from family and friends, lower psychological distress, and a harmonious parent–child relationship all lead to a better coping strategy for the affected parents. This invariably carries over into the care of the patients [6, 28, 38].

In a 2009 study, Bos et al. determined the OHRQoL of Dutch orthodontic patients and their parents. They presented lower values for the socioemotional and the well-being subscales in the girls group as compared to the boys group [11]. This contradicts the results of our present study. In the descriptive analysis of gender, the girls showed higher COHIP scores in total and in the three subscales, though without statistical significance. This result is in line with the studies by Kramer et al. [41] and van Roy et al. [74]. Feragen and Stock conducted a psychological evaluation of patients with CL/P at the ages of 10 and 16 years. They determined that male patients at 10 years of age showed lower psychological adjustment than females, while the reverse became true at the age of 16 years [23, 24]. We can conclude that, in terms of gender, psychological adjustment is dependent on age. The results of the COHIP total score of the older age group of 12–18 years showed significantly lower values in group 1. We can assume that the older patients are more self-reflective, observing, and comparing themselves more with pubertal development. Puberty was the criteria to split the age groups in this study between 11 and 12 years.

The second hypothesis was rejected for group 1. Speech and esthetic concerns seem to have been important factors affecting the health-related quality of life for children with CL/P [20, 58]. These factors seem to be more important as children approach adolescence (ages 8–12 years), when acceptance by peers becomes more critical. Chetpakdeechit et al. determined that during childhood patients with CLP are not as aware of their condition, feeling more like children without craniofacial disorders. As they grow older, these patients become aware of their malformation. Their concerns included the following: being treated differently, appearing different, lack of recognition, and wanting most of all to be treated like children without a malformation [17]. Growing older increased the importance of the OHRQoL, with patients suffering from craniofacial disorders expressing the negative side of their appearance. This can lead to developing functional, social, emotional, and speech problems during the transition from childhood to adolescence [3].

In our study, patients with CL ± A showed the highest OHRQoL, followed by those with CP and those with a unilateral cleft. The validity of this result has to be regarded carefully due to the small case number of patients with CL ± A and RS. RS and bilateral clefts had the lowest COHIP total score values. Dulfer et al. described the HRQoL in RS children [21]. Parents of RS patients reported a lower HRQoL than parents of children unaffected by RS. This was due to respiratory problems such as upper airway obstruction of the patients with RS. In contrast, a study of Basart et al. demonstrated that the HRQoL in patients with RS was comparable with an unaffected control group, although parental distress was higher in the syndromic RS group than in the nonsyndromic group [8]. In the study of Basart et al., no significant difference was determined in the visibility of the cleft. This is in line with findings from the current literature [23, 24].

Many studies have reported on patient assessments with CL/P based on questionnaires, interviews, or observations by self-report or others [13, 30, 47, 51, 59, 65]. In our study, we used the COHIP-G19 questionnaire to evaluate OHRQoL. Sierwald et al. proved that the German version of COHIP-G19 is a sufficient tool in assessing psychometric properties in children and adolescents [64]. The test score reliability coefficient, or Cronbach’s α, to evaluate internal consistency of the COHIP-SF19 questionnaire provided good values for nearly all subscales. Only the functional and socioemotional subscales of group 2 revealed unacceptable values. This might be due to the fact that, regarding group 1 versus group 2, group 1 shows better values of internal consistency, suggesting that patients with craniofacial disorder may be more reliable in their answers. Furthermore, the COHIP-G19 questionnaire is designed for patients with craniofacial health problems and has proven reliable specifically for these patients. For investigations concerning children and the effects of dental treatments, or in epidemiologic studies on oral health outcome, the use of condition-specific QOL measures like the COHIP has the advantage of increased patient responsiveness, since the assessment is focused on a specific condition, namely oral health, and it involves increased sensitivity to treatment effects.

Only a few interdisciplinary cleft teams routinely carry out regular psychological assessment, as recommended by the American Cleft Palate Craniofacial Association [28]. Adequate interdisciplinary therapy from a multidisciplinary team that is always interested in improvement is the basis of successful treatment for these complex and sensitive patients who are in rehabilitation throughout their early years, and even longer. The results of this study reveal that an early start in oral hygiene instruction and prevention, speech therapy, prosthetic and conservative rehabilitation, and psychological support—not only for the affected patients but for the entire family—are imperative, making up a fundamental part of therapy in general. These findings are similar to those published by Feragen and Stock [23]. Overall, the aim of therapy for patients with craniofacial disorder is optimizing function in terms of feeding, eating, speech, and hearing, as well as achieving the best esthetic results while also providing social and socioemotional support to patients and their families, especially before patients begin school [42]. Only by looking at all these factors can we reduce the development of deeper social and emotional problems and the risk of bullying or social exclusion. The constant improvement of therapy is essential in raising awareness on how to identify and deal with these patients and to improve rehabilitation and patient care.

### Study limitations and outlook

The small sample size limits the power of this study. The smaller the sample size, the more difficult it becomes to predict the meaningfulness of the received COHIP scores. To compare reliably patients with RS versus nonsyndromic patients with CLP, the sample size of the RS population needs to be increased. To assess the impact of the craniofacial disorder on the QoL within the family environment of the affected patient, one could use the Coping Health Inventory for Parents (CHIP).

In reviewing the current literature, we found several gaps in the fields of cleft care [34, 53]. Significantly lower values in the oral-health subscales for patients with craniofacial malformation compared to the healthy control group are a limiting factor. Future studies need to address the effects of orthodontic treatment or secondary alveolar bone grafting on OHRQoL. For this purpose, patients should answer in future studies the COHIP-G19 questionnaire before and after treatment in order to acquire comparative results. The subject matter of this study is interesting across many disciplines, regardless of the location. Thus, a multicenter study would be critical for future research as a means of producing a longitudinal approach in clarifying differences in protocols.

## Conclusion

This study revealed that the presence of a craniofacial disorder is an important factor in the OHRQoL of affected patients. Patients without craniofacial disorders statistically had a significantly better OHRQoL than patients with a disorder. Female patients with a craniofacial disorder had a better OHRQoL than male patients, though this difference was not statistically significant. Males with a craniofacial disorder scored significantly lower than those without a disorder. Patients experienced a lower OHRQoL as they grew older, independent of the presence of a craniofacial disorder. The significantly lower COHIP scores for oral-health, pronunciation, bullying, and confidence subscales show that early dental and rehabilitation treatment, speech training, and psychological care are necessary for improved overall treatment of patients. Furthermore, it becomes obvious that pronunciation and speech continue to be problematic for patients with craniofacial disorders and additional explorations are required.

Understanding the influence of a craniofacial disorder on the OHRQoL will help to guide health-care professionals in raising awareness of such factors and identifying affected patients and their families. This may advance the opportunities in specific interdisciplinary treatment. A future multicenter study with other cleft centers would be critical in furthering a longitudinal approach.
